# Effect of Photodynamic Therapy on Gemcitabine-Resistant Cholangiocarcinoma *in vitro* and *in vivo* Through KLF10 and EGFR

**DOI:** 10.3389/fcell.2021.710721

**Published:** 2021-11-03

**Authors:** Yang Yang, Jigang Li, Lei Yao, Lile Wu

**Affiliations:** ^1^Department of Clinical Pathology, Hunan Cancer Hospital, Changsha, China; ^2^Department of Hepatobiliary Surgery, The Affiliated Hospital of Southwest Medical University, Luzhou, China; ^3^Academician Expert Workstation of Sichuan Province, The Affiliated Hospital of Southwest Medical University, Luzhou, China

**Keywords:** cholangiocarcinoma, gemcitabine resistance, photodynamic therapy (PDT), KLF10, EGFR, cell cycle 3

## Abstract

Cholangiocarcinoma is a relatively rare neoplasm with increasing incidence. Although chemotherapeutic agent such as gemcitabine has long been used as standard treatment for cholangiocarcinoma, the interindividual variability in target and drug sensitivity and specificity may lead to therapeutic resistance. In the present study, we found that photodynamic therapy (PDT) treatment inhibited gemcitabine-resistant cholangiocarcinoma cells *via* repressing cell viability, enhancing cell apoptosis, and eliciting G1 cell cycle arrest through modulating Cyclin D1 and caspase 3 cleavage. *In vivo*, PDT treatment significantly inhibited the growth of gemcitabine-resistant cholangiocarcinoma cell-derived tumors. Online data mining and experimental analyses indicate that KLF10 expression was induced, whereas EGFR expression was downregulated by PDT treatment; KLF10 targeted the EGFR promoter region to inhibit EGFR transcription. Under PDT treatment, EGFR overexpression and KLF10 silencing attenuated the anti-cancer effects of PDT on gemcitabine-resistant cholangiocarcinoma cells by promoting cell viability, inhibiting apoptosis, and increasing S phase cell proportion. Importantly, under PDT treatment, the effects of KLF10 silencing were significantly reversed by EGFR silencing. In conclusion, PDT treatment induces KLF10 expression and downregulates EGFR expression. KLF10 binds to EGFR promoter region to inhibit EGFR transcription. The KLF10/EGFR axis participates in the process of the inhibition of PDT on gemcitabine-resistant cholangiocarcinoma cells.

## Introduction

Cholangiocarcinoma is a diverse group of malignancies arising from the biliary epithelium and a relatively rare neoplasm in developed countries; however, the incidence of cholangiocarcinoma is increasing globally (Siegel et al., 2019). Due to the difficulty of prognostic accuracy, at least half of patients are diagnosed with unresectable tumors and progress to an advanced stage (Blechacz and Gores, 2008; Kim et al., 2017). Thus, advanced, or metastatic disease patients present an overall survival of less than 6 months and a 5-year survival rate of less than 10% (Marin et al., 2018).

Systemic chemotherapy, as well as single-agent molecular targeted therapy are the conventional treatments for cholangiocarcinoma. For example, gemcitabine, one of the most widely used chemotherapeutic drugs for treating cholangiocarcinoma, is a nucleoside deoxycytidine analog that can enter cells *via* nucleoside receptors and then activate deoxycytosine kinases to bind DNA (Thongprasert, 2005; Alvarellos et al., 2014; Fan et al., 2017). Gemcitabine leads to apoptosis *via* blocking cell cycle progression at the G1/S phase boundary (Plunkett et al., 1995; Gilbert et al., 2006; Fan et al., 2017). However, patients with advanced cholangiocarcinoma often obtain chemoresistance and show poor response to chemotherapy (Park et al., 2015; Morizane et al., 2019). For example, patients with inoperable cholangiocarcinoma received gemcitabine therapy only obtained relatively low 5-year survival rates (Valle et al., 2010; Razumilava and Gores, 2013; Rizvi and Gores, 2013). The interindividual variability in target and drug sensitivity and specificity may lead to therapeutic resistance. According to the understanding of the cell mechanism related to cholangiocarcinoma growth and drug reaction, multimodal therapies including combined treatment have emerged as a reasonable method to promote the therapeutic efficacy.

Photodynamic therapy (PDT) employs light activation of tissue-localized photosensitizer in an oxygen-dependent process (the most convenient light source is a laser). In the first stage of PDT, a tumor selective photosensitizer is administered (Dougherty et al., 1998; Bown et al., 2002), followed after some time by the illumination with visible light, which, in the presence of oxygen, leads to the generation of cytotoxic species and consequently to cell death and tissue destruction (Dougherty et al., 1998; Dolmans et al., 2003; Ayaru et al., 2007). PDT has been reported to be used upon skin lesions or hollow organ walls, and in recent years more attention has been paid to its potential in the treatment of solid organ lesions and digestive tract dysplasia and early cancerous lesions (Evrard et al., 1991; Schuitmaker et al., 1996; Bown et al., 2002), including cholangiocarcinoma (Kahaleh, 2012; McCaughan et al., 1991; Talreja et al., 2011). Although the synergetic anti-tumor effects of PDT/gemcitabine combination have been reported within cholangiocarcinoma (Chen et al., 2014; Hong et al., 2014; Wentrup et al., 2016; Kim et al., 2018), the mechanism underlying the synergetic anticancer effect remains unclear.

Previous studies indicated that PDT might induce cancer cell survival pathway activation. For example, within perihilar cholangiocarcinoma (QBC939) cells, sublethal PDT (LC50) led to the alteration of survival signaling pathways such as HIF-1, NF-κB, AP-1, and heat shock factor (HSF) (Luna et al., 1994; Broekgaarden et al., 2015; Weijer et al., 2015, 2016). PDT-treated QBC939 cell line also exhibited decreased protein levels related to the EGFR pathway, especially at LC90 (Weijer et al., 2017). Notably, HIF-1 induction within Het-1a, a human esophageal squamous epithelial cell line, decreased 5-ALA-PDT-induced cell death and apoptosis; the pro-survival response of HIF-1 showed to be inhibited after siRNA-mediated knockdown of HIF-1, thereby increasing PDT efficacy within the Het-1a cell line (Ji et al., 2006). Verteporfin-PDT induced EGFR and STAT3 expression in OVCAR-5 and H460 cancer cells, whereas the EGFR or STAT-3 silencing with siRNA augmented PDT efficacy (Edmonds et al., 2012). Thus, identifying PDT-targeted survival pathways might provide an in-depth understanding of the synergetic anticancer effect of PDT and gemcitabine.

Herein, the study firstly constructed cholangiocarcinoma cells with resistance to gemcitabine and examined the specific effects of PDT exposure on regular and gemcitabine-resistant cholangiocarcinoma cell viability, apoptosis, and cell cycle distribution. The *in vivo* effects of PDT treatment on regular and gemcitabine-resistant cholangiocarcinoma cell-derived implanted tumor growth were also investigated. Then, bioinformatics analyses were performed using online datasets to identify differentially expressed transcription factors and genes after PDT treatment and KLF10 and EGFR were found. The predicted binding and regulation between KLF10 and EGFR were verified, and the specific effects of KLF10 and EGFR upon the synergetic anti-tumor effects of PDT/gemcitabine combination were examined, respectively and combinedly. In conclusion, we identified transcription factors and signaling that might participate in PDT reversing cholangiocarcinoma resistance to gemcitabine.

## Materials and Methods

### Cell Resource

The human cholangiocarcinoma cell line RBE and QBC939 was obtained from Xiangya Cell Bank (Changsha, China) and cultured in RPMI1640 medium (Invitrogen, Carlsbad, CA, United States) supplemented with 10% FBS (Invitrogen). All cells were cultured at 37°C in 5% CO_2_.

### Induction of Gemcitabine-Resistant Cholangiocarcinoma Cell Lines

RBE and QBC939 cells were exposed to the graded concentrations of gemcitabine, as described previously (De Angelis et al., 2004). Generally, a total of 1 × 10^5^ cells was cultured in 25-cm^2^ flasks for 24 h and exposed to gemcitabine (0.25, 0.5, 1, 2, 4, 8, 16, 32, and 64 μM) for 72 h. Surviving cells were cultured in drug-free medium to allow cells to attain 80% confluence. Then, the cells were cultured at this drug concentration until they grew steadily and the IC50 values were determined by the MTT assay. These surviving cells were then exposed to gemcitabine at twofold increase of IC50 concentration for six rounds. Two gemcitabine-resistant cell lines, RBE-R and QBC939-R were obtained after 8 months of culture. All two gemcitabine-resistant cell lines were grown in drug-free medium for 2 weeks then harvested, frozen in the liquid nitrogen, and stored at −80°C until analyzed.

### MTT for Cell Viability

Cells were seeded in 96-well plates at a density of 5 × 10^3^ cells/well and treated with gemcitabine for 72 h or PDT treatment. Cell viability was evaluated with the 3-(4,5-dimethylthiazol-2yl)-2,5-diphenyltetrazolium bromide (MTT) assay (AMRESCO, Solon, OH, United States). Half-maximal inhibitory concentration (IC50) was analyzed relative to the DMSO control. Values are shown as the means of triplicate wells from three independent experiments for each drug concentration.

### Flow Cytometry for Cell Cycle and Cell Apoptosis

For cell cycle analysis, cells were plated in 6-well plates at a density of 1 × 10^5^ cells/well for 24 h. After treatment and/or transfection, cells were harvested, washed twice with cold PBS, fixed overnight in 70% ethanol at 4°C, incubated for 30 min in the dark with RNase A and propidium iodide (PI) (final concentration 2.4 μg/ml) at room temperature. The cell cycle distribution was examined using an ACEA NovoCyte flow cytometer (Becton-Dickinson, San Jose, CA, United States), and the data were analyzed using FlowJo software.

For cell apoptosis, an Annexin V-FLUOS staining kit (Roche Diagnostic, Mannheim, Germany) was used. Cells were plated in 6-well plates at a density of 1 × 10^5^ cells/well for 24 h. After treatment and/or transfection, floating and adherent cells were collected, washed twice with cold PBS, resuspended in 100 μl binding buffer containing 2 μl Annexin V-FITC and 2 μl PI (50 μg/ml) and incubated at room temperature for 15 min in the dark. Then, cell apoptosis was analyzed using a FACScanto^TM^ II flow cytometer (Becton-Dickinson), and the data were analyzed using FACS Diva^TM^ software (Becton-Dickinson).

## Immunoblotting

Target cells were lysed with the iced hypotonic buffer. After estimating the protein concentrations, the samples containing the proteins were loaded and separated on SDS–PAGE. Then, the blots were transferred to a PVDF membrane and incubated with the primary antibodies for 24 h at 4°C. The following antibodies were used: anti-ki67 (27309-1-AP; Proteintech, Wuhan, China), anti-Cyclin D1 (60186-1-Ig, Proteintech), anti-cleaved-caspase 3 (ab2302; Abcam, Cambridge, MA, United States), anti-caspase 3 (19677-1-AP, Proteintech), anti-EGFR (CSB-PA10279A0Rb; CUSABIO, Houston, TX, United States), Erk1/2 (67170-1-Ig, Proteintech), p-Erk1/2 (sc-81492; Santa Cruz, Dallas, TX, United States), anti-Akt (Y409094; ABM, Richmond, BC, Canada), anti-p-Akt (Y011054, ABM), anti-VEGF (CSB-PA07529A0Rb, CUSABIO), anti-KLF10 (ab73537, Abcam), and anti-GAPDH (ab8245, Abcam). After that, the membrane was incubated with HRP-conjugated secondary antibody (1:1000) for 1 h at 37°C. The visualization of the proteins was achieved by the enhanced chemiluminescence (ECL) reagent.

### Subcutaneously Implanted Tumor Model in Nude Mouse

Gemcitabine-resistant QBC939-R/REB-R or gemcitabine-sensitive QBC939/REB cells were implanted into 5-week-old BALB/c nude mice. Briefly, the cells (2 × 10^6^) in 100 μl of serum-free RPMI were injected subcutaneously into the flank of the mice. Mice bearing human cholangiocarcinoma cells-derived xenograft tumors were treated or non-treated with PDT. Tumor size and tumor weight were measured every 3 days, and volumes were determined using the formula volume = length × width^2^/2. At day 35, the mice were sacrificed. The tumor tissues were collected and the protein levels of ki67, CyclinD1, and cleaved-caspase3/caspase3 were examined in tumor tissues. All procedures were approved by the Institutional Animal Care and Use Committee (IACUC) of The Affiliated Hospital of Southwest Medical University in accordance with the “Principles of Laboratory Animal Care” (NIH, Bethesda, MD, United States).

### Mice Photodynamic Therapy

After tumor diameter of nude mice reached over 6 mm for about 15 days, mice bearing QBC939/REB- or QBC939-R/REB-R-derived tumors were randomized to divide into 8 groups, including QBC939 (*n* = 6), QBC939 + PDT (*n* = 6), QBC939-R (*n* = 6), QBC939-R + PDT (*n* = 6), REB (*n* = 6), REB + PDT (*n* = 6), REB-R (*n* = 6), REB-R + PDT (*n* = 6). Mice received intratumoral administration of 20% 5-ALA (Sigma Chemical Co.) at a dose of 100 mg/kg, following by PDT for 5 min at 4 h later (630 nm, 100 J/cm^2^, 100 mW/cm^2^). The diameter of the irradiating laser beam entirely covered the tumor. At 14 days after irradiation, the mice were sacrificed. Tumor sizes and weights were analyzed statistically.

### RT-qPCR

RNA was extracted from target cells and cDNA was prepared using the oligo-dT-based Transcriptor first-strand cDNA synthesis kit (Roche Diagnostics, Basel, Switzerland) with an input of 500 ng total RNA according to the manufacturer’s instructions and diluted in RNAse-free H_2_O to obtain a final concentration of 5 ng/μl. The RT-qPCR assays were performed using a Power SYBR Green PCR Master Mix (Life Technologies, Carlsbad, CA, United States) in an ABI Prism 7900HT instrument (Applied Biosystems, Carlsbad, CA, United States). The relative expression levels were calculated using the 2^–Δ^ ^Δ^ ^*Ct*^ method, taking the GAPDH mRNA level as an internal reference.

### Bioinformatics Analysis

The expression and clinical data were acquired from The Cancer Genome Atlas (TCGA)-cholangiocarcinoma (CHOL) data and the Gene Expression Omnibus (GEO) data with accession numbers GSE84756 and GSE68292. The GSE84756 dataset includes the whole genome expression profiling of biliary adenocarcinoma cells (SK-ChA-1) while were treated with the buffer (control group), dark toxicity (DT group), 50% lethal concentration of 500-mW laser light (LC50) group) or super lethal concentration of 500-mW laser light (LC90 group). The GSE68292 dataset includes the gene expression of hilar cholangiocarcinoma (SK-ChA-1) cells which were treated with PBS (control group), dark (0 mW group), 50-mW laser light (50 mW group), or 500-mW laser light (500 mW group). The TCGA-CHOL dataset includes the gene expression data of 9 normal control tissues and 36 cholangiocarcinoma tissues. The online tool UALCAN (Chandrashekar et al., 2017)^1^ was used for KLF10 expression, overall survival, disease-specific survival and progression-free interval analysis. The analysis for the microarrays GSE84756 and GSE68292 were analyzed by R language Limma package (Ritchie et al., 2015) with the condition of | log2 (fold change)| ≥ 0.56, *P* < 0.05.

### Chromatin Immunoprecipitation Assay

Chromatin immunoprecipitation assays were performed by using the Magna ChIP Kit (Millipore, Bedford, MA, United States) following the manufacturer’s directions. QBC939 cells were treated with formaldehyde to generate DNA-protein cross-links. Cell lysates were sonicated to generate chromatin fragments of 200–300 bp, and the lysates were immunoprecipitated with anti-KLF10 or anti-IgG (internal reference). The precipitated chromatin DNA was recovered and measured by qPCR.

### Dual-Luciferase Reporter Assay

To verify the binding between KLF10 and the EGFR promoter, we generated psicheck2-proEGFR and psicheck2-proEGFR-mut luciferase reporter plasmids. The plasmids were then co-transfected into 293T cells with a negative control vector or KLF10-overexpressing vector (vector/KLF10); 48 h after the transfection, the luciferase activity was determined using the Dual-Luciferase Assay Kit (Promega, ıMadison, WI, United States).

### Statistical Analysis

Statistical analyses were performed using GraphPad (GraphPad Software, Inc., La Jolla, CA, United States). Significant differences between groups were evaluated by a Student’s *t*-test. The results are reported as the means ± standard deviation (SD) based on at least three replicates.

## Results

### Effects of Photodynamic Therapy on Cholangiocarcinoma Gemcitabine Resistance

**FIGURE 1:**
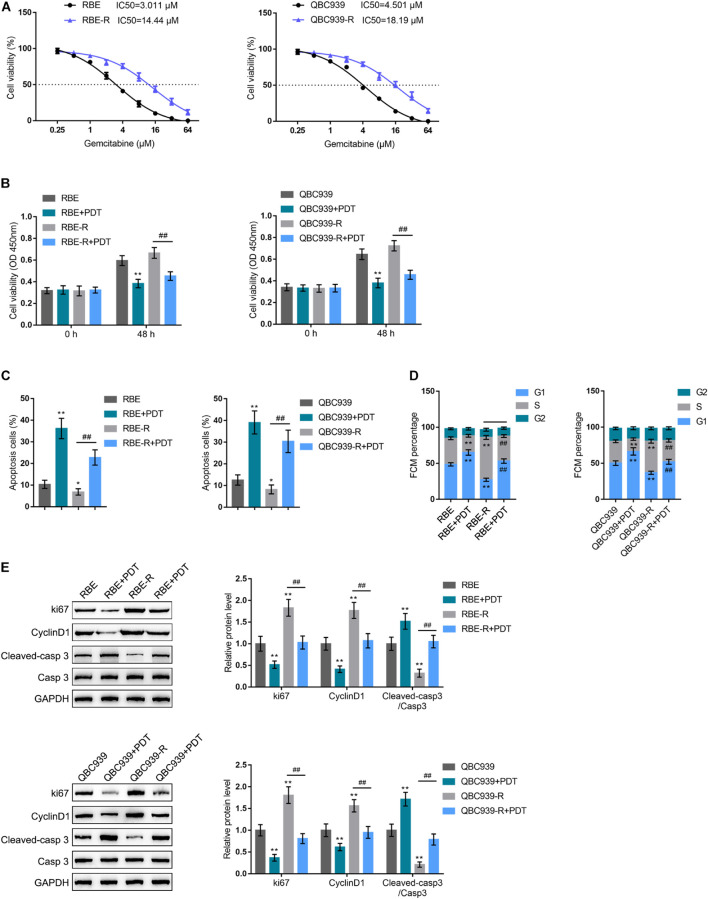
Effects of PDT on gemcitabine-resistant cholangiocarcinoma cells. **(A)** Gemcitabine-resistant cholangiocarcinoma cell lines, RBE-R and QBC939-R were established as described; the cell viability was determined by MTT assays and shown as the IC50 values. **(B)** Regular and gemcitabine-resistant cholangiocarcinoma cell lines were exposed or non-exposed to PDT treatment and examined for the cell viability by MTT assays. Then, RBE, QBC939, RBE-R, and QBC939-R cells were exposed or non-exposed to PDT treatment and examined for the cell apoptosis by Flow cytometry **(C)**; cell cycle distribution by Flow cytometry **(D)**; the protein levels of ki67, Cyclin D1, cleaved-caspase 3, and caspase 3 by Immunoblotting **(E)**. *P < 0.05, **P < 0.01, ^##^*P* < 0.01.

To investigate the specific functions of PDT exposure upon cholangiocarcinoma gemcitabine resistance to gemcitabine, we firstly established two gemcitabine-resistant cholangiocarcinoma cells, RBE-R and QBC939-R, as described. After 8 months induction, the IC50 values of regular RBE and QBC939 cell lines were increased from 3.011, 4.501 to 14.44, 18.19 μM, respectively (Figure 1A), suggesting that the gemcitabine-resistant cancer cells were successfully constructed. Secondly, these cholangiocarcinoma cells were exposed or non-exposed to PDT treatment and examined for the cell viability. Figure 1B showed that RBE-R, and QBC939-R cell viability was higher than that of regular RBE, and QBC939 cell; PDT exposure significantly inhibited the cell viability of all cancer cells and RBE-R, and QBC939-R cell viability more inhibited. Then, RBE-R and QBC939-R cell lines showed to be exposed or non-exposed to PDT treatment and examined for the cell apoptosis and cell cycle distribution. Compared with RBE and QBC939 cell line, respectively, the apoptosis of RBE-R and QBC939-R cell line was significantly inhibited; PDT significantly promoted apoptosis of these cell lines (Figure 1C and Supplementary Figure 1A). Compared with RBE and QBC939 cells, respectively, RBE-R and QBC939-R cell cycle tended to distribute in S phase; PDT exposure induced cell cycle in G1 phase within these cell lines (Figure 1D and Supplementary Figure 1B). As for the proliferating and apoptotic markers, the protein levels of ki67 and Cyclin D1 were significantly increased, whereas cleaved-caspase 3/caspase 3 was decreased in RBE-R and QBC939-R cells; PDT exposure significantly reversed the changes in these proteins in these cell lines (Figure 1E). These data suggest that PDT exposure exhibited cytotoxicity on regular RBE and QBC939 cell line and gemcitabine-resistant RBE-R and QBC939-R cell line.

### Photodynamic Therapy Treatment Inhibits Gemcitabine-Resistant Cholangiocarcinoma Cells Xenotransplanted Tumors

**FIGURE 2:**
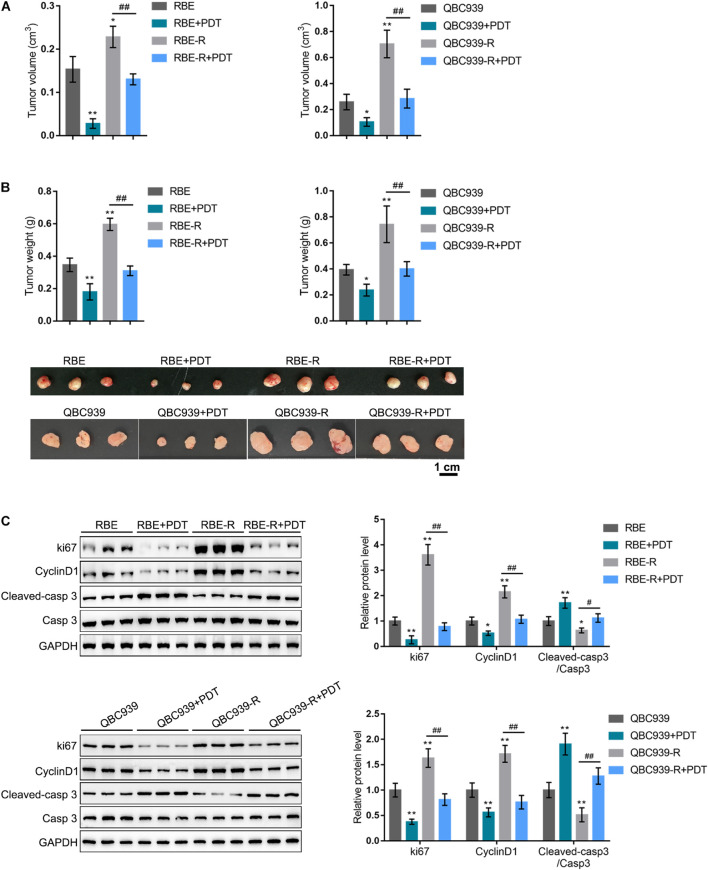
PDT treatment inhibits gemcitabine-resistant cholangiocarcinoma cells xenotransplanted tumors. **(A,B)** Xenotransplanted tumor model derived from regular RBE and QBC939 or gemcitabine-resistant RBE-R and QBC939-R cells was established in nude mice as described in section “Materials and Methods.” Twenty-eight days after transplanting, the tumors were undergone PDT treatment. Fourteen days later, the tumor size was measured and the tumor volume was calculated **(A)**; the tumor weight was measured **(B)**; **(C)** the protein levels of ki67, Cyclin D1, cleaved-caspase 3, and caspase 3 in tumor tissues were examined by Immunoblotting. *P < 0.05, **P < 0.01, ^##^*P* < 0.01.

To further confirm the *in vitro* findings, we established a xenotransplanted tumor model in nude mice by injecting regular RBE and QBC939 cell lines or gemcitabine-resistant RBE-R and QBC939-R cell lines. Mice bearing tumors derived from regular RBE and QBC939 cell line or gemcitabine-resistant RBE-R and QBC939-R cell line showed to be treated or non-treated with PDT. Twenty-eight days after transplanting, the tumor volume (Figure 2A) and tumor weight (Figure 2B) of RBE-R or QBC939-R-derived tumors were significantly larger than those of the RBE or QBC939-derived tumors, respectively. For both types of tumors, PDT treatment significantly reduced the tumor volume and tumor weight (Figures 2A,B). Moreover, the protein levels of ki67, Cyclin D1, cleaved-caspase 3, and caspase 3 were examined in tumor tissues. Consistent with *in vitro* findings, ki67 and Cyclin D1 proteins showed to be dramatically increased, whereas cleaved-caspase 3/caspase 3 was decreased in RBE-R or QBC939-R-derived tumors, compared with those in RBE or QBC939-derived tumors, respectively (Figure 2C). PDT exposure significantly reversed the alterations in these proteins in both types of tumors (Figure 2C). These *in vivo* findings suggest that PDT treatment exhibits anti-tumor effects on gemcitabine-resistant cell-derived tumors.

### Identification of Differentially Expressed Genes That Could Participate in Photodynamic Therapy Reversing the Resistance of Cholangiocarcinoma Cells to Gemcitabine

Transcription factors are at the core of gene expression regulation. To investigate the mechanism underlying PDT reversing cholangiocarcinoma gemcitabine resistance, we performed online data mining to identify transcription factors altered by PDT treatment by bioinformatics analyses. GSE84756 and GSE68292 datasets were compared, and found that these two datasets intersected in 31 differentially expressed transcription factors (11 downregulated and 20 upregulated) within PDT-subjected QBC939 cell line (Supplementary Table 1). Among the 20 upregulated transcription factors, CEBPD, CSRNP1, and KLF10 were regularly underexpressed in cholangiocarcinoma but upregulated by PDT treatment. According to TCGA- CHOL data, KLF10 expression was significantly downregulated in cholangiocarcinoma tissues (Supplementary Figure 2A). Moreover, also according to TCGA-CHOL data, cholangiocarcinoma patients with lower KLF10 expression predicted poorer overall survival (Supplementary Figure 2B), disease-specific survival (Supplementary Figure 2C), and progression-free survival (Supplementary Figure 2D). Thus, KLF10 might participate in the process of PDT reversing cholangiocarcinoma gemcitabine resistance.

**FIGURE 3:**
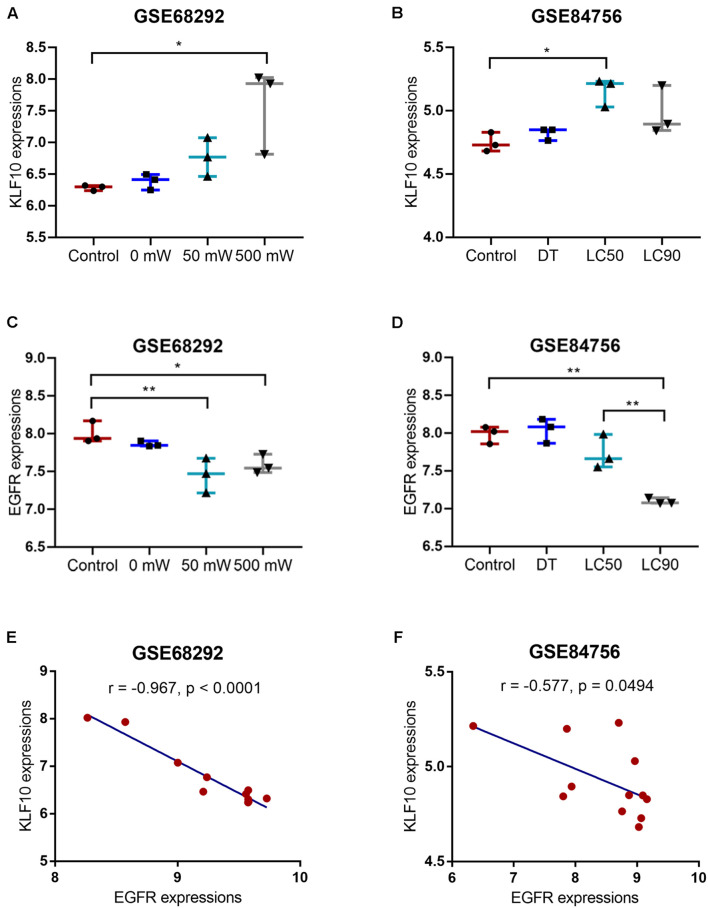
KLF10 expression and correlation with EGFR. **(A)** KLF10 expression under the control, 0, 50, or 500 mW PDT exposure, according to GSE68292. **(B)** KLF10 expression under the control, dark toxicity (DT), LC50, or LC90 PDT exposure, according to GSE84756. **(C)** EGFR expression under the control, 0, 50, or 500 mW PDT exposure, according to GSE68292. **(D)** EGFR expression under the control, dark toxicity (DT), LC50, or LC90 PDT exposure, according to GSE84756. **(E)** The correlation between KLF10 and EGFR expression, according to GSE68292. **(F)** The correlation between KLF10 and EGFR expression, according to GSE84756. *P < 0.05, **P < 0.01.

As for the downstream signaling involved, also based on GSE84756 and GSE68292, a total of 147 genes were upregulated and 181 downregulated after PDT treatment. These deregulated genes were applied for DAVID KEGG signaling enrichment annotation, and 17 were enriched in tumor signaling, including 11 downregulated (EGFR, AXIN2, WNT7B, ADCY1, GNG12, GNAI1, ITGB1, ITGA6, LAMB1, LAMC1, and RUNX1) and 6 upregulated (CXCL8, FOS, MYC, JUN, NFKB2, and TRAF4) by PDT. Then, KLF10 targeted signaling was predicted and EGFR was among the KLF10 targeted genes. According to GSE84756 and GSE68292, KLF10 expression was significantly upregulated, whereas EGFR expression was downregulated by PDT treatment (Figures 3A–D). In tissue samples, KLF10 and EGFR expression were negatively correlated (Figures 3E,F). Thus, we hypothesize that KLF10 might target the EGFR promoter region to inhibit EGFR transcription. KLF10 and EGFR might be involved in the process of PDT reversing cholangiocarcinoma gemcitabine resistance.

### KLF10 Inhibits EGFR Transcription by Binding the Promoter Region

**FIGURE 4:**
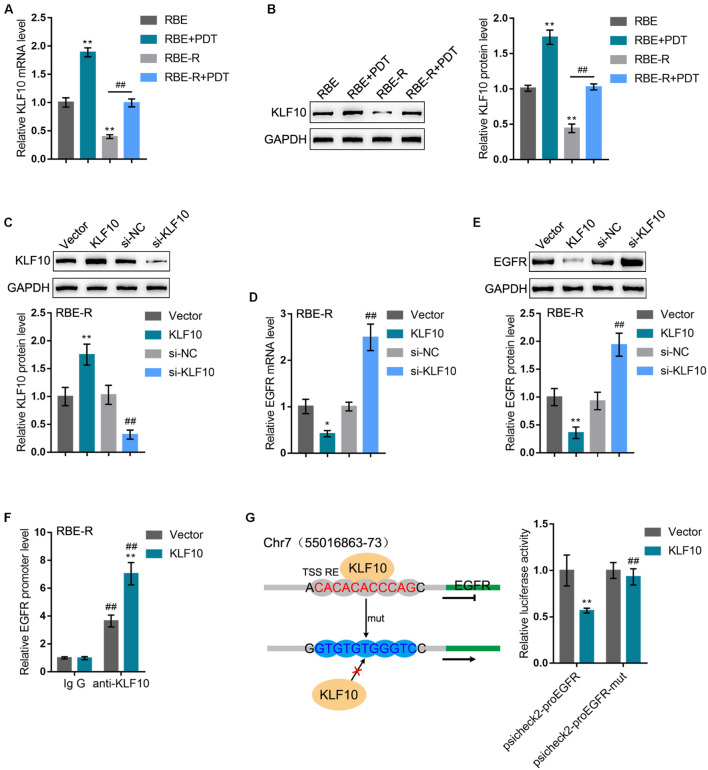
KLF10 inhibits EGFR transcription by binding the promoter region. **(A)** RBE and RBE-R cells were treated or non-treated with PDT and examined for KLF10 mRNA expression by real-time qPCR. **(B)** RBE and RBE-R cells were treated or non-treated with PDT and examined for KLF10 protein levels by Immunoblotting. **(C)** KLF10 overexpression or silencing was achieved in RBE-R cells by transfecting KLF10-overexpressing vector or small interfering RNA targeting KLF10. The transfection efficiency was verified using Immunoblotting. Then, RBE-R cells were transfected with KLF10 or si-KLF10 and examined for the mRNA expression of EGFR by real-time qPCR **(D)** and the protein levels of EGFR by Immunoblotting **(E)**. **(F)** RBE-R cells were transfected with KLF10 or empty vector and ChIP assay was performed using anti-KLF10 or anti-IgG (negative control). The EGFR promoter levels in the immunoprecipitate was examined using real-time qPCR. **(G)** Wild-type psicheck2-proEGFR and mutant type psicheck2-proEGFR-mut luciferase reporter plasmids were constructed and co-transfected in 293T cells with KLF10 or empty vector. The luciferase activity was examined by a dual-luciferase reporter assay. *P < 0.05, **P < 0.01, ^##^*P* < 0.01.

Before investigating the roles of KLF10 and EGFR in PDT reversing cholangiocarcinoma gemcitabine resistance, we first examined the predicted KLF10 binding and negative regulation of EGFR. RBE and gemcitabine-resistant RBE-R cell line showed to be exposed or non-exposed to PDT treatment and examined for KLF10 mRNA expression and protein levels. KLF10 expression was downregulated within RBE-R cells compared with RBE cells. Moreover, consistent with online microarray expression profiles, PDT treatment significantly induced KLF10 mRNA expression and protein levels in both REB and REB-R cells (Figures 4A,B). Then, KLF10 overexpression or silencing was achieved in RBE-R cells by transfecting KLF10-overexpressing vector or small interfering RNA targeting KLF10. The transfection efficiency was verified using Immunoblotting (Figure 4C). In RBE-R cells, EGFR mRNA expression and protein levels were significantly downregulated by KLF10 overexpression, whereas upregulated by KLF10 silencing (Figures 4D,E). These data confirmed that KLF10 negatively regulates EGFR expression.

To confirm the binding of KLF10 to EGFR promoter, we transfected RBE-R cells with KLF10 or empty vector and performed ChIP assay using anti-KLF10 or anti-IgG (negative control). We employed real-time qPCR to examine the EGFR promoter levels in the immunoprecipitate. Figure 4F showed that the EGFR promoter levels were significantly increased within the immunoprecipitate of anti-KLF10 compared with that in anti-IgG group; moreover, KLF10-overexpressing cells obtained higher EGFR promoter levels compared with that in cells transfected with empty expression vector. Next, dual-luciferase reporter assay was performed by constructing wild-type psicheck2-proEGFR and mutant type psicheck2-proEGFR-mut luciferase reporter plasmids and co-transfected them in 293T cells with KLF10 or empty vector. Figure 4G showed that KLF10 overexpression dramatically repressed psicheck2-proEGFR plasmid luciferase activity; when co-transfected with psicheck2-proEGFR-mut, KLF10 overexpression failed to alter the luciferase activity. In summary, KLF10 could inhibit EGFR transcription *via* directly binding to the EGFR promoter region.

### EGFR Might Participate in Photodynamic Therapy Reversing Cholangiocarcinoma Gemcitabine Resistance

**FIGURE 5:**
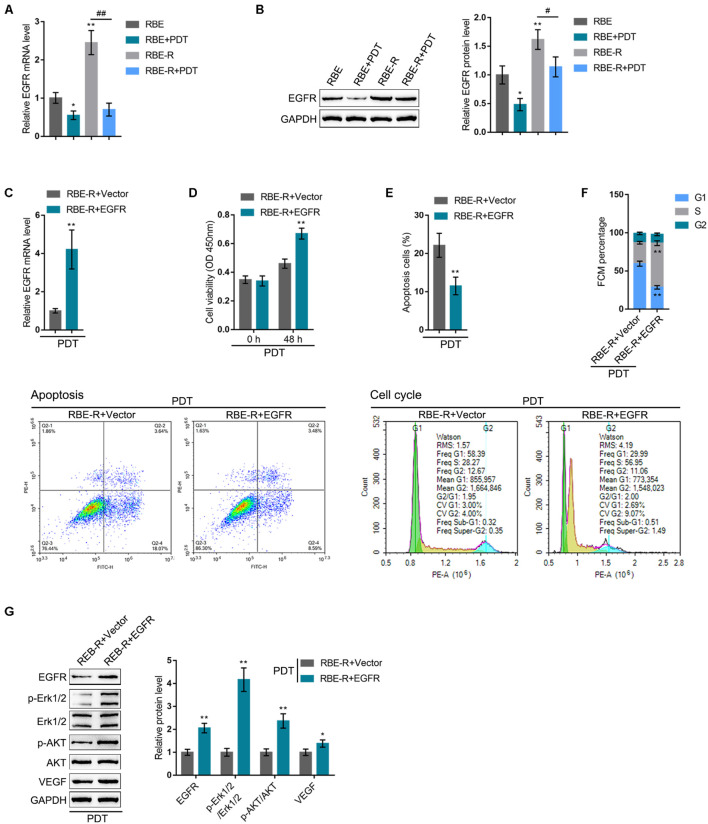
EGFR might participate in PDT reversing cholangiocarcinoma gemcitabine resistance. RBE or gemcitabine-resistant RBE-R cells were treated or non-treated with PDT and examined for EGFR mRNA expression by real-time qPCR **(A)** and the protein levels of EGFR by Immunoblotting **(B)**. **(C)** EGFR overexpression was achieved in RBE-R cells by transfecting EGFR-overexpressing vector (EGFR). The transfection efficiency was verified using real-time qPCR. Then, RBE-R cells were transfected with EGFR or empty vector (negative control), exposed to PDT treatment, and examined for cell viability by MTT assay **(D)**; cell apoptosis by Flow cytometry **(E)**; cell cycle distribution by Flow cytometry **(F)**; the protein levels of EGFR, Erk1/2, p-Erk1/2, Akt, p-Akt, and VEGF by Immunoblotting **(G)**. *P < 0.05, **P < 0.01, ^#^*P* < 0.05, ^##^*P* < 0.01.

After confirming KLF10 negative regulation of EGFR, next, the specific effects of these two factors on the process of PDT reversing cholangiocarcinoma gemcitabine resistance were investigated. RBE or gemcitabine-resistant RBE-R cell lines showed to be exposed or non-exposed to PDT treatment and examined for EGFR mRNA and protein expression. EGFR mRNA and protein expression showed to be significantly upregulated within RBE-R cell line compared with RBE cell line; PDT treatment significantly downregulated the mRNA and protein expression of EGFR within these two cell lines (Figures 5A,B). Next, EGFR overexpression was achieved within RBE-R cell line under PDT treatment by transfecting the EGFR-overexpressing vector (EGFR). The transfection efficiency was verified using real-time qPCR (Figure 5C). Under PDT treatment, EGFR overexpression significantly promoted RBE-R cell viability (Figure 5D), inhibited cell apoptosis (Figure 5E), and increased the proportion of S phase cells (Figure 5F). As for the proliferating and apoptotic markers, under PDT treatment, EGFR overexpression significantly increased EGFR and VEGF proteins, as well as the ratio of p-Erk1/2/Erk1/2 and p-Akt/Akt (Figure 5G). These data suggest that EGFR overexpression could attenuate gemcitabine-resistant cholangiocarcinoma cell response to PDT treatment.

### KLF10 Silencing Attenuates the Effects of Photodynamic Therapy on Gemcitabine-Resistant Cells

**FIGURE 6:**
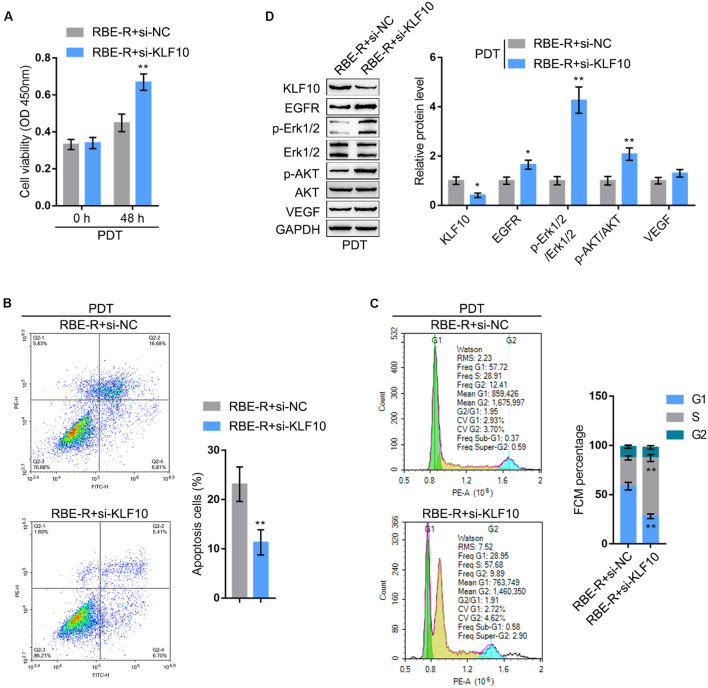
KLF10 silencing attenuates the effects of PDT on gemcitabine-resistant cells. RBE-R cells were transfected with si-KLF10 or si-NC (negative control), exposed to PDT treatment, and examined for the cell viability by MTT assay **(A)**; cell apoptosis by Flow cytometry **(B)**; cell cycle distribution by Flow cytometry **(C)**; the protein levels of KLF10, EGFR, p-Erk1/2, Erk1/2, p-Akt, Akt, and VEGF by Immunoblotting **(D)**. *P < 0.05, **P < 0.01.

Considering that PDT significantly induces KLF10 expression, next, KLF10 silencing was achieved in RBE-R cells under PDT treatment by transfecting si-KLF10 or si-NC (negative control). Similar to EGFR overexpression, under PDT treatment, KLF10 silencing significantly promoted RBE-R cell viability (Figure 6A), repressed cell apoptosis (Figure 6B), and increased the proportion of S phase cells (Figure 6C). As for the proliferating and apoptotic markers, under PDT treatment, KLF10 silencing decreased the protein levels of KLF10, whereas increased EGFR and VEGF protein levels and the ratio of p-Erk1/2/Erk1/2 and p-Akt/Akt significantly (Figure 6D).

### KLF10 Modulates Photodynamic Therapy Reversing Cholangiocarcinoma Gemcitabine Resistance Through EGFR

**FIGURE 7:**
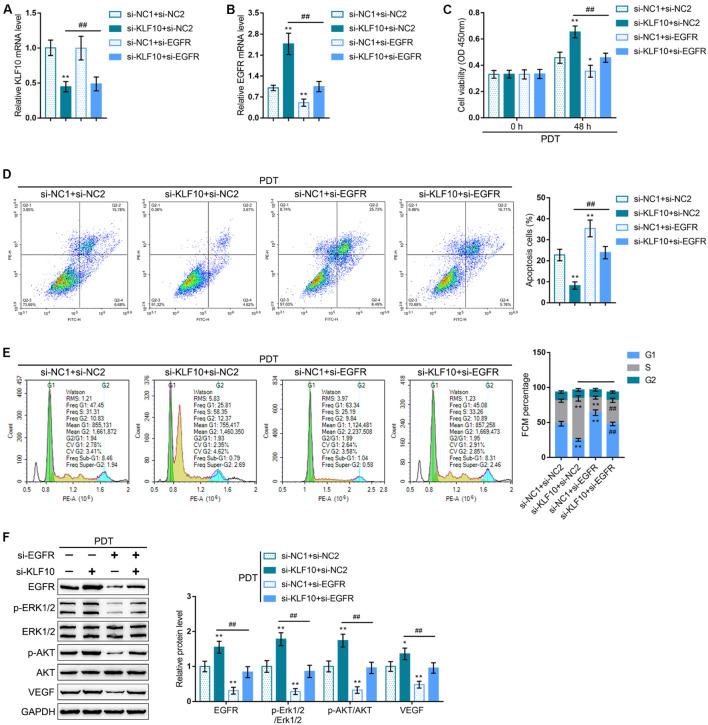
KLF10 modulates PDT reversing cholangiocarcinoma gemcitabine resistance through EGFR. RBE-R cells were co-transfected with si-KLF10 and si-EGFR, exposed to PDT treatment, and examined for the mRNA expression of KLF10 **(A)** and EGFR **(B)** by real-time qPCR; cell viability by MTT assay **(C)**; cell apoptosis by Flow cytometry **(D)**; cell cycle distribution by Flow cytometry **(E)**; the protein levels of KLF10, EGFR, p-Erk1/2, Erk1/2, p-Akt, Akt, and VEGF by Immunoblotting **(F)**. *P < 0.05, **P < 0.01, compared with the si-NC1 + si-NC2 group; ^##^*P* < 0.01, compared with the si-KLF10 + si-NC2 group.

To investigate whether the KLF10/EGFR axis plays a dynamic role in the process of PDT reversing cholangiocarcinoma gemcitabine resistance, we co-transfected RBE-R cells with si-KLF10 and si-EGFR, exposed the cells to PDT treatment, and examined for the mRNA expression of KLF10 and EGFR. As shown in Figures 7A,B, under PDT treatment, si-KLF10 transfection downregulated KLF10 expression and upregulated EGFR expression, and si-EGFR transfection caused no alteration in KLF10 expression and downregulated EGFR expression; the effects of si-KLF10 transfection on EGFR expression was significantly reversed by si-EGFR transfection (Figures 7A,B). As for the cellular functions, KLF10 silencing promoted cell viability, repressed cell apoptosis, and increased the proportion of S phase cells (Figures 7C–E); on the contrary, EGFR silencing inhibited cell viability, enhanced cell apoptosis, and elicited G1 cell cycle arrest (Figures 7C–E). The effects of KLF10 silencing on cell phenotype were reversed by EGFR silencing (Figures 7C–E). As for the proliferating and apoptotic markers, KLF10 silencing increased EGFR and VEGF proteins and the ratio of p-Erk1/2/Erk1/2 and p-Akt/Akt (Figure 7F); on the contrary, EGFR silencing reduced EGFR and VEGF proteins and the ratio of p-Erk1/2/Erk1/2 and p-Akt/Akt (Figure 7F). The effects of KLF10 silencing on these markers were reversed by EGFR silencing.

## Discussion

In the present study, we found that PDT treatment inhibited gemcitabine-resistant cholangiocarcinoma cells *via* repressing cell viability, enhancing cell apoptosis, and eliciting G1 cell cycle arrest through modulating Cyclin D1 and caspase 3 cleavage. *In vivo*, PDT treatment significantly inhibited the growth of gemcitabine-resistant cholangiocarcinoma cell-derived tumors. Online data mining and experimental analyses indicate that KLF10 expression was induced, whereas EGFR expression was downregulated by PDT treatment; KLF10 targeted the EGFR promoter region to inhibit EGFR transcription. Under PDT treatment, EGFR overexpression and KLF10 silencing attenuated the anti-cancer effects of PDT on gemcitabine-resistant cholangiocarcinoma cells by promoting cell viability, inhibiting apoptosis, and increasing S phase cell proportion. Importantly, under PDT treatment, the effects of KLF10 silencing were significantly reversed by EGFR silencing.

The use of PDT with concomitant chemotherapy is frequently seen in the treatment of cancers. Nevertheless, limited data are available to analyze PDT combined with chemotherapy. Several prospective and retrospective studies were performed comparing the outcome of PDT combined with chemotherapy vs. PDT alone (Hong et al., 2014; Park et al., 2014; Wentrup et al., 2016). Another retrospective study was performed to compare the overall survival of PDT combined with chemotherapy vs. chemotherapy alone, and PDT/chemotherapy combination was found to present the potential of survival benefits, nevertheless, the difference did not reach statistically significant (*P* = 0.47) (Knuppel et al., 2012). Gonzalez-Carmona et al. (2019) first indicated that PDT/chemotherapy combination could lead to an obviously higher overall survival in patients with unresectable cholangiocarcinoma than chemotherapy alone (*P* = 0.022). Herein, the study constructed gemcitabine-resistant cholangiocarcinoma cells and found that PDT exposure indeed inhibited these cells, as manifested as inhibited cell viability, enhanced cell apoptosis, and G1 cell cycle arrest. Moreover, PDT treatment inhibited the growth of the tumor derived from gemcitabine-resistant cholangiocarcinoma cells. Despite previous prospective and retrospective studies and our present findings, the specific molecular mechanism underlying the synergetic anti-tumor effects of PDT/gemcitabine combination remain unclear.

Indeed, PDT treatment could alter a range of survival pathways, including HIF-1, NF-κB, AP-1, HSF, and EGFR (Luna et al., 1994; Broekgaarden et al., 2015; Weijer et al., 2015, 2016, 2017). In the present study, by cross-checking online microarray expression profiles reporting PDT-altered genes, we found that KLF10, a transcription factor, could be significantly induced by PDT treatment, which was further evidenced by the observation that PDT treatment upregulated KLF10 mRNA expression and protein levels in gemcitabine-resistant cholangiocarcinoma cells. The KLF transcription factor family performs a variety of biological functions (McConnell and Yang, 2010), and several members of KLF family are found to be associated with certain aspects of tumor cell biology, such as cell growth, cell apoptosis, cell differentiation and cell migration (Tetreault et al., 2013; Cheng et al., 2019; Zhang et al., 2020). KLF10 enhances human leukemia cell death by upregulating Bim and Bax pro-apoptotic proteins (Jin et al., 2007). Within cholangiocarcinoma, KLF10 could be regulated by miR-106b and might participate in the anti-apoptotic effects of miR-106b on cholangiocarcinoma cells (Wehrkamp et al., 2018). Thus, KLF10 might be involved in the inhibition of PDT on gemcitabine-resistant cholangiocarcinoma cells.

Considering that transcription factors are at the core of gene expression regulation, we also searched for possible target genes of KLF10. Notably, EGFR was in the intersection of KLF10 target genes and PDT treatment-downregulated genes. EGFR was overexpressed in multiple cancer types (Herbst and Shin, 2002), such as perihilar cholangiocarcinoma (Harder et al., 2009; Yang et al., 2014), and was affected by PDT using liposome bound ZnPc (Weijer et al., 2017). EGFR is an emerging therapeutic target for treating cancers, and the approval of monoclonal cetuximab and panitumumab and the kinase inhibitors gefitinib and erlotinib is evidence of this (Joseph et al., 2012). In the present study, EGFR mRNA expression and protein levels in gemcitabine-resistant cholangiocarcinoma cells were significantly downregulated by PDT treatment. Since KLF10 targets the promoter region of EGFR and inhibits EGFR transcription, we speculate that KLF10 might play a role in the synergetic anti-tumor effects of PDT/gemcitabine combination through inhibiting EGFR.

Gemcitabine was phosphorylated into gemcitabine monophosphate by deoxycytidine kinase (dCK) after an influx of nucleoside transporters into cell membranes, which underwent a complex intracellular transformation to gemcitabine diphosphate (dFdCDP) and triphosphate (dFdCTP), responsible for its cytotoxicity, thereby leading to inhibition of DNA synthesis and induction of apoptosis *via* blocking cell cycle progression at the G1/S phase boundary (Heinemann et al., 1995; Plunkett et al., 1995; Galmarini et al., 2002). As we observed in the present study, PDT treatment on gemcitabine-resistant cholangiocarcinoma cell lines elicited G1 cell cycle arrest, repressed cell viability, and enhanced cell apoptosis through modulating the cell cycle regulator and apoptosis-associated Cyclin D1 and caspase 3. As speculated, KLF10 silencing or EGFR overexpression attenuated the anti-cancer effects of PDT on gemcitabine-resistant cholangiocarcinoma cells by increasing S phase cell proportion, promoting cell viability, and inhibiting cell apoptosis. More importantly, when co-transfected to gemcitabine-resistant cholangiocarcinoma cells, the effects of si-KLF10 were significantly reversed by EGFR silencing, indicating that KLF10 participates in the inhibition of PDT on gemcitabine-resistant cholangiocarcinoma cells through EGFR.

## Conclusion

In conclusion, PDT treatment induces KLF10 expression and downregulates EGFR expression. KLF10 binds to the EGFR promoter region to inhibit EGFR transcription. The KLF10/EGFR axis participates in the process of the inhibition of PDT on gemcitabine-resistant cholangiocarcinoma cells growth. These occurrences forebode that PDT treatment could be deemed as a newfangled strategy for the treatment of gemcitabine chemoresistance cholangiocarcinoma. However, in clinical chemotherapy of cholangiocarcinoma, gemcitabine and platinum-based drug combination is the first-line treatment (Abdel-Rahman et al., 2018). Multidrug resistance is one of the major challenges in cholangiocarcinoma treatment (Gonzalez-Carmona et al., 2019). Therefore, the function and mechanism of PDT in multidrug-resistant cholangiocarcinoma need to be investigated in the future.

## Data Availability Statement

The original contributions presented in the study are included in the article/Supplementary Material, further inquiries can be directed to the corresponding author.

## Ethics Statement

All procedures were approved by the Institutional Animal Care and Use Committee (IACUC) of The Affiliated Hospital of Southwest Medical University in accordance with the “Principles of Laboratory Animal Care” (NIH, Bethesda, MD, United States).

## Author Contributions

LW designed the study and critically revised the manuscript. YY led the animal experiments and drafted the manuscript. JL and LY performed experiments on animal and cells, collected the samples, and analyzed the samples and data. All authors edited the manuscript and approved the final manuscript.

## Conflict of Interest

The authors declare that the research was conducted in the absence of any commercial or financial relationships that could be construed as a potential conflict of interest.

## Publisher’s Note

All claims expressed in this article are solely those of the authors and do not necessarily represent those of their affiliated organizations, or those of the publisher, the editors and the reviewers. Any product that may be evaluated in this article, or claim that may be made by its manufacturer, is not guaranteed or endorsed by the publisher.

## Footnotes

1
http://ualcan.path.uab.edu/index.html

## Supplementary Material

Supplementary Figure 1 Effects of PDT on gemcitabine-resistant cholangiocarcinoma cells apoptosis and cycle. RBE, QBC939, RBE-R, and QBC939-R cells were exposed or non-exposed to PDT treatment and examined for the cell apoptosis by Flow cytometry **(A)**; cell cycle distribution by Flow cytometry **(B)**.

Supplementary Figure 2 Correlation of KLF10 expression with the prognosis of patients with cholangiocarcinoma. **(A)** KLF10 expression in normal (*n* = 9) and cholangiocarcinoma (*n* = 36) tissues, according to TCGA-cholangiocarcinoma (CHOL) database. Then, cases from TCGA-CHOL were divided into high and low KLF10 expression groups using the median value of KLF10 expression as cut-off, and the correlation between KLF10 expression and the overall survival **(B)**, disease-specific survival **(C)**, progression-free interval **(D)** in patients with cholangiocarcinoma was analyzed using a Cox proportional hazard regression model and log-rank analysis. Results were shown as Kaplan–Meier curves.

Supplementary Table 1 Differentially expressed transcription factors in cholangiocarcinoma QBC939 cells treated by PDT based on GSE84756 and GSE68292.
