# Supplementary figures and images for: Effect of Photodynamic Therapy on Gemcitabine-Resistant Cholangiocarcinoma in vitro and in vivo Through KLF10 and EGFR
Source: Front Cell Dev Biol. 2021 Nov 3;9:710721. doi: 10.3389/fcell.2021.710721 (PMC8595284; doi:10.3389/fcell.2021.710721)

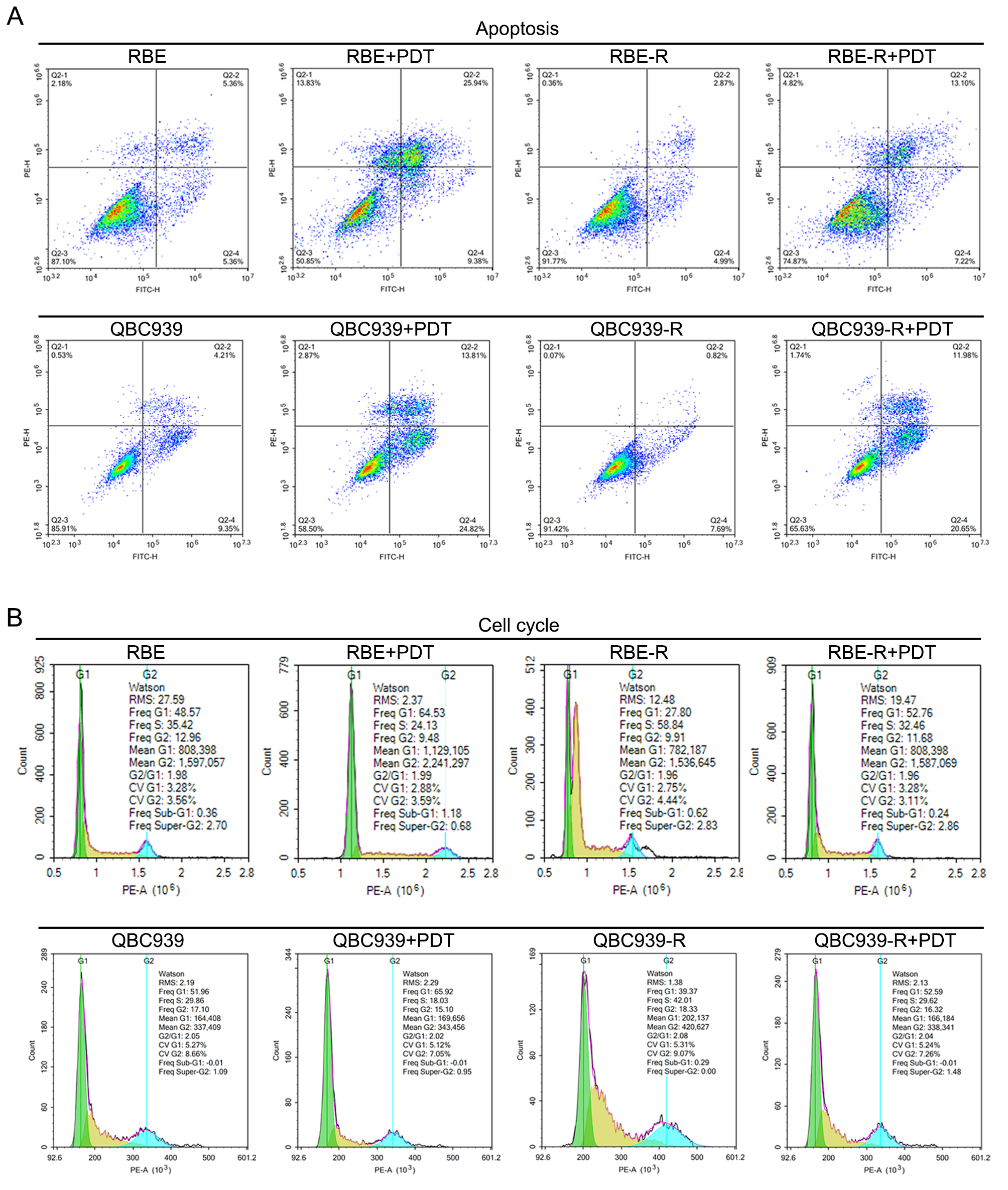

Supplement: Supplementary Figure 1 — Effects of PDT on gemcitabine-resistant cholangiocarcinoma cells apoptosis and cycle. RBE, QBC939, RBE-R, and QBC939-R cells were exposed or non-exposed to PDT treatment and examined for the cell apoptosis by Flow cytometry (A); cell cycle distribution by Flow cytometry (B). [file Image_1.TIF]

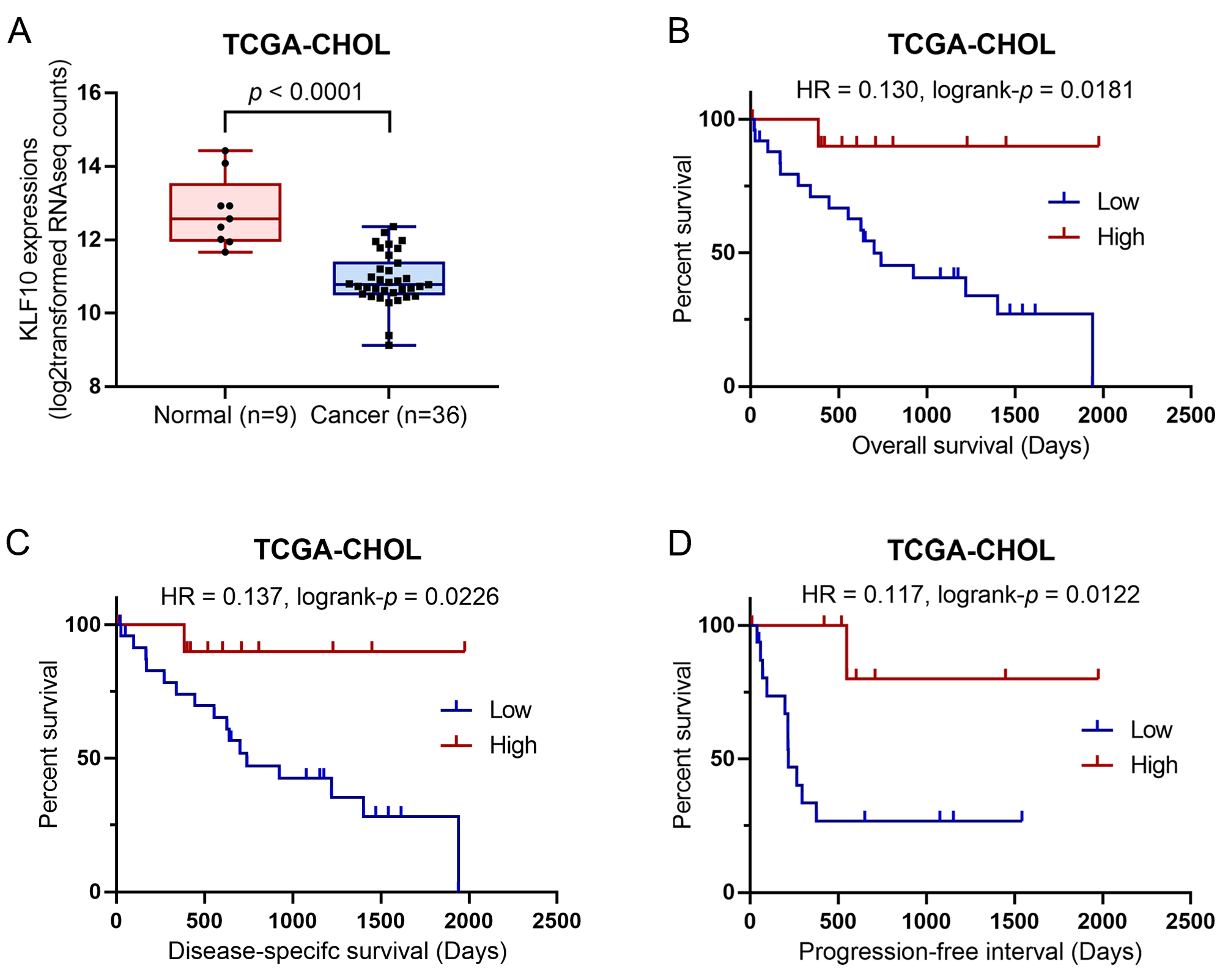

Supplement: Supplementary Figure 2 — Correlation of KLF10 expression with the prognosis of patients with cholangiocarcinoma. (A) KLF10 expression in normal (n = 9) and cholangiocarcinoma (n = 36) tissues, according to TCGA-cholangiocarcinoma (CHOL) database. Then, cases from TCGA-CHOL were divided into high and low KLF10 expression groups using the median value of KLF10 expression as cut-off, and the correlation between KLF10 expression and the overall survival (B), disease-specific survival (C), progression-free interval (D) in patients with cholangiocarcinoma was analyzed using a Cox proportional hazard regression model and log-rank analysis. Results were shown as Kaplan–Meier curves. [file Image_2.TIF]
