# Supplementary material for: Effect of Photodynamic Therapy on Gemcitabine-Resistant Cholangiocarcinoma in vitro and in vivo Through KLF10 and EGFR
Source: Front Cell Dev Biol. 2021 Nov 3;9:710721. doi: 10.3389/fcell.2021.710721 (PMC8595284; doi:10.3389/fcell.2021.710721)
Supplement: Supplementary Table 1 — Differentially expressed transcription factors in cholangiocarcinoma QBC939 cells treated by PDT based on GSE84756 and GSE68292. [file Table_1.docx]

**Table S1 Differentially expressed transcription factors in cholangiocarcinoma QBC939 cells treated by PDT based on GSE84756 and GSE68292**

| **NCBI gene ID** | **Gene name** | **Regulation after PDT in two GEO sets** | **ID** |
| --- | --- | --- | --- |
| 467 | ATF3 | Up | ILMN_1791346 |
| 1052 | CEBPD | Up | ILMN_1782050 |
| 253782 | CERS6 | Down | ILMN_1767662 |
| 64764 | CREB3L2 | Down | ILMN_1751097 |
| 64651 | CSRNP1 | Up | ILMN_1703123 |
| 1958 | EGR1 | Up | ILMN_1762899 |
| 1959 | EGR2 | Up | ILMN_1743199 |
| 2117 | ETV3 | Up | ILMN_1703180 |
| 2353 | FOS | Up | ILMN_1669523 |
| 2354 | FOSB | Up | ILMN_1751607 |
| 8061 | FOSL1 | Up | ILMN_1771841 |
| 57801 | HES4 | Up | ILMN_1653466 |
| 9592 | IER2 | Up | ILMN_1700584 |
| 3665 | IRF7 | Up | ILMN_2349061 |
| 3720 | JARID2 | Down | ILMN_1764177 |
| 3725 | JUN | Up | ILMN_1806023 |
| 7071 | KLF10 | Up | ILMN_2411897 |
| 1316 | KLF6 | Up | ILMN_1735014 |
| 23764 | MAFF | Up | ILMN_1680139 |
| 4609 | MYC | Up | ILMN_1680618 |
| 4791 | NFKB2 | Up | ILMN_2390859 |
| 4800 | NFYA | Up | ILMN_1690325 |
| 5013 | OTX1 | Down | ILMN_1691180 |
| 861 | RUNX1 | Down | ILMN_1801504 |
| 6591 | SNAI2 | Up | ILMN_2082585 |
| 27324 | TOX3 | Down | ILMN_2413833 |
| 10194 | TSHZ1 | Down | ILMN_1718907 |
| 114821 | ZBED9 | Down | ILMN_2246154 |
| 7543 | ZFX | Down | ILMN_1687484 |
| 7549 | ZNF2 | Down | ILMN_1687884 |
| 7637 | ZNF84 | Down | ILMN_3300358 |
